# Assessment of heart rate variability and occurrence of falls in Alzheimer's disease: an exploratory study

**DOI:** 10.1055/s-0045-1814401

**Published:** 2026-01-25

**Authors:** Evelize Antunes Rodrigues, Aline Roberta Danaga, Etiene Farah Teixeira de Carvalho, Carlos Alberto Santos Filho, José Burgos Ponce, Alessandro Ferrari Jacinto

**Affiliations:** 1 Universidade Estadual Paulista, Faculdade de Medicina de Botucatu, Botucatu SP, Brazil.; 2 Universidade Federal de Alfenas, Alfenas MG, Brazil.; 3 Universidade Nove de Julho, São Paulo SP, Brazil; 4 Centro Universitário de Adamantina, Adamantina SP, Brazil.

**Keywords:** Heart Rate Monitoring, Alzheimer Disease, Autonomic Nervous System, Dementia

## Abstract

**Background:**

Aging is accompanied by an increasing incidence of dementia. Alzheimer's disease (AD) is the leading cause of dementia, and it impairs autonomic function. Heart rate variability (HRV) is a marker of autonomic function, but findings in AD are conflicting, and there is scant information on the association of HRV with falls in dementia patients.

**Objective:**

To assess autonomic activity in older adults with AD, comparing these patients to older adults without dementia (the control group, CG), and to investigate the relationship between HRV and falls.

**Methods:**

The HRV was analyzed in older adults without dementia and in those with AD using a heart rate monitor. The measurements were made on a single day in the supine and orthostatic positions for 10 minutes each. The HRV components in the time and frequency domains were assessed, along with the history of falls in the past 3 years.

**Results:**

The groups were homogenous, with a predominance of female individuals, and mean ages of 81 (AD) and 79 (CG) years. A reduction in the R-R interval upon changing from the supine to the orthostatic positions was evident in both groups, but the AD group showed reduced parasympathetic components in the orthostatic position. For the frequency domain, a reduction in high frequency (HF) and increases in low frequency (LF) and in the LF/HF ratio were observed, suggesting increased sympathetic and reduced parasympathetic activities. The AD group presented more falls, whose incidence was associated with HRV components.

**Conclusion:**

Alzheimer's disease was associated with worse autonomic dysfunction, increased sympathetic activity and greater parasympathetic impairment, a high incidence of falls and interaction with HRV components.

## INTRODUCTION

Aging is accompanied by an increase in the prevalence of dementia, 1 with the risk of developing the disease doubling every 5 years after the age of 65 years, reaching 25% at the age 80 years. 2 Dementia causes cognitive and behavioral impairments, with the condition often associated with underlying Alzheimer's disease (AD), 3 vascular dementia, frontotemporal Lewy bodies, and Parkinson's disease. 4 5

The leading cause of dementia (60% of the cases), AD accounted for 35 million cases globally in 2013, a figure set to double by 2030 and to triple by 2050, to reach a total of 115.4 million cases worldwide. 6 7

Alzheimer's disease is characterized by the accumulation of extracellular Beta-amyloid and intracellular hyperphosphorylated Tau, besides possible associations with cardiovascular risk factors. 8 This condition commences in the hippocampus and entorhinal cortex, initially impairing recent memory. As AD progresses, it later impacts other cognitive domains, behavior, and overall functioning. 4 In this context, autonomic dysfunction, involving sympathetic and parasympathetic injuries, frequently occurs, 5 8 9 leading to reduced physical activity and mobility, impaired cardiovascular conditioning, and a heightened risk of falls. This risk is frequently exacerbated by the use of psychotropic agents. 8 10 11

Considering autonomic dysfunction, a previous study 12 comparing older adults with AD to healthy controls demonstrated subtly impaired parasympathetic activity and relative sympathetic overactivity in the dementia group, which may nonetheless contribute to distinctive functional and cognitive disturbances.

This autonomic impairment can be studied through an analysis of the heart rate variability (HRV), which determines low-frequency (LF) and high-frequency (HF) power bands from the successive differences between heartbeat intervals, reflecting sympathetic and parasympathetic activation, respectively, on sinoatrial node activity. 13

Thus, increased sympathetic and reduced parasympathetic activities raise the cardiovascular risk, whereas a high HRV index indicates better autonomic health. 14 Conversely, low HRV is a strong risk indicator of adverse events, 15 and it is routinely used in the analysis of hypertension, myocardial infarction, heart failure, diabetes, and neurodegenerative diseases, including AD. 16

Given its high prevalence and severe impact on functional capacity, dementia represents a major public health concern associated with a high socioeconomic burden. 17 Moreover, the risk of falls is eight times higher in older adults with dementia. 18 Although the clinical connection is evident, few studies have explored the association between HRV and falls in AD, 5 16 including limited Brazilian research, 10 12 and even fewer have used portable heart rate monitors for this assessment. 19 Therefore, the current study aimed to assess autonomic activity in older adults with dementia due to AD and explore the possible interaction between HRV and falls.

## METHODS

We conducted an analytical cross-sectional study involving older adults. The study was previously approved by the local Ethics in Research Committee (under CAAE: 55442416.5.0000.5411), and all participants signed the free and informed consent form.

### Inclusion criteria

We included older adults aged ≥ 60 years, who were able to walk and stand without assistance. The participants with AD comprised individuals who were clinically diagnosed with the disease according to their medical records, based on criteria for the diagnosis of dementia and AD in Brazil, 3 whereas the control group (CG) included subjects who were cognitively healthy, as measured by the Mini-Mental State Examination (MMSE). All participants continued to use their medications normally.

### Exclusion criteria

In the AD group, individuals with behavioral problems which hindered assessment or who were at advanced stages of the disease were excluded. The following exclusion criteria were applied to both groups: major clinical/hemodynamic abnormalities, atrial fibrillation or other arrhythmias, presence of a heart pacemaker, and difficulties which hampered signal collection.

The participants with AD were assessed at health centers and community centers in the municipality of Botucatu (state of São Paulo, Brazil), whereas the cognitively-healthy participants were assessed in the municipalities of Botucatu and Avaré. The investigation included anamnesis, cognitive assessment using the MMSE, and analysis of the HRV for cardiovascular autonomic control. Data were collected from the older adults, their caregivers or medical records, and included sociodemographic, anthropometric and clinical variables, besides history of falls. The level of physical activity was self-reported, and sedentary lifestyle was defined as engagement in fewer than 150 minutes of physical exercise per week. Based on the MMSE score, the cognitive impairment status was classified into mild (18–23 points) or severe (0–17 points). 20 21

The HRV recordings were performed in a quiet environment, as per the guidelines of the Task Force on Heart Rate Variability. 22 The participants were given guidance prior to the exam, and they were evaluated in a temperature-controlled room. A Polar heart rate monitor (Polar Electro Oy) was used to record the heart rate and R-R intervals (RRis), with collection performed for 10 minutes in the supine position and for another 10 minutes in the orthostatic position. The data gathered were analyzed using the Kubios HRV Scientific (Kubios Oy) analysis software, version 2 β, for stationary segments selected for the spectral analysis of the HRV, expressed in milliseconds squared (ms ^2^ ) and in normalized units (nu). An autoregressive spectral model was applied to calculate LF (0.04 - 0.15 Hz) and HF power bands (0.15 - 0.4 Hz), as well as the LF/HF ratio, representing the sympathovagal balance index.

## RESULTS

We initially analyzed 35 older men and women. The analysis of autonomic profiles yielded satisfactory results for 26 participants, 11 without dementia, who formed the control group (CG), and 15 participants with AD, who formed the case group. The final sample was reached after the exclusion of cases during the analyses, predominantly due to interference in the transmitted signals (6 cases) and restlessness when placed in the decubitus position (3 cases).

The CG and AD groups had similar mean ages and no significant differences regarding weight, height, or body mass index. The participants were predominantly white female subjects. Tobacco and alcohol use was more common in the AD group, although this was not statistically significant ( Table 1 ). There were similar frequencies of comorbidities, and the AD participants presented cases of arthrosis, osteoporosis, stroke, heart murmur, and liver cirrhosis, besides a greater level of sedentary lifestyle. One CG participant was considered active according to the recommendations of the World Health Organization (WHO), and five participants (4 in the CG and 1 in the AD group) engaged in irregularly in physical activities. The cognitive status (according to the MMSE) was significantly lower in the AD group, and the mean time since the diagnosis of dementia was of 4.3 years. Most participants had 5 to 8 years of schooling in the CG and 1 to 4 years in the AD group, without correlation between the MMSE and HRV variables.

**Table 1:** General and clinical baseline characteristics of the participants (N = 26)

		CG (n = 11)	AD group (n = 15)	*p* *
Mean age (years)		79 ± 7.55	81 ± 6.80	0.44
Mean weight (kilos)		68.2 ± 12.94	65.6 ± 14.22	0.63
Mean height (meters)		1.59 ± 0.10	1.54 ± 0.07	0.41
Mean BMI (kg/m ^2^ )		21.4 ± 3.23	21.3 ± 4.42	0.94
Skin color (white): n (%)		7 (64%)	10 (67%)	1.00
Female sex: n (%)		9 (82%)	8 (73%)	0.36
Smoking: n (%)		0	3 (20%)	0.24
Alcohol consumption: n (%)		0	1 (7%)	1.00
Sedentary lifestyle: n (%)		6 (54%)	14 (93%)	**0.05**
**Clinical conditions: n (%)**	Hypertension	8 (73%)	13 (87%)	0.69
	Diabetes mellitus	2 (18%)	5 (33%)	0.66
	Hypothyroidism	3 (27%)	2 (13%)	0.62
	Mean MMSE score	27 ± 3.1	15 ± 6.7	**< 0.001**
**Medication use: n (%)**	Antihypertensive	9 (82%)	10 (67%)	0.06
	Beta-blockers	2 (18%)	3 (20%)	1.00
	Diuretics	5 (45%)	8 (53%)	0,71
	Anxiolytics	4 (36%)	5 (33%)	1.00
	Antidepressants	1 (9%)	3 (20%)	0.61
	Neuroleptics agents	0	7 (47%)	**0.01**
	Gastrointestinal protectors	1 (9%)	8 (53%)	**0.03**
	Insulin	0	1 (6%)	1.00
	Memantine	1 (9%)	7 (47%)	**0.08**
	Anticholinesterase drugs	1 (9%)	6 (40%)	**0.18**

Abbreviations: AD, Alzheimer's disease; BMI, body mass index; CG, control group; MMSE, Mini-Mental State Examination.

Notes: *Student's *t-* test for continuous variables and Chi-squared or Fisher's exact test for categorical variables; values in bold indicate statistical significance.

The frequency of medication use was similar between the groups for most therapeutic classes ( Table 1 ). Conversely, the AD participants reported significantly higher use of antipsychotic agents and gastrointestinal protectors. The use of memantine and anticholinesterase drugs was also higher in this group, though these differences did not reach statistical significance.

The results of the HRV analysis revealed significantly greater RRis in the AD group for the supine and orthostatic positions ( *p* = 0.005 and 0.0127 respectively). Other parameters showed no significant group differences, but a tendency for higher numerical values was evident in the AD participants. The results of the HRV analysis in the frequency domain, measured in the supine and orthostatic positions for both groups, are presented in Table 2 , and Table 3 provides a comparison of HRV components in the supine and orthostatic positions for the two groups.

**Table 2:** Heart rate variability frequency domains for the supine and orthostatic positions

		CG (n =1 1)	AD group (n = 15)	*p* *
Supine position	VLF (ms ^2^ )	11 (9–19)	24 (12.5–54.5)	**0.004**
	LF (ms ^2^ )	30 (22.5–42.5)	69 (46.5–120.5)	**0.027**
	HF (ms ^2^ )	18 (10.5–54.5)	48 (23–114.5)	0.203
	LF (nu)	52.38 (35.4–71.9)	60.67 (42.1–75.4)	0.451
	HF (nu)	47.40 (27.8–63.8)	39.15 (24.5–57.7)	0.516
	LF/HF ratio	1.11 (0.58–2.59)	1.55 (0.7–3.1)	0.483
Orthostatic position	VLF (ms ^2^ )	11 (5–21.5)	28 (11.5–50.5)	**0.038**
	LF (ms ^2^ )	37 (21.5–82)	88 (31.5–157.0)	0.264
	HF (ms ^2^ )	17 (5.0–32.5)	15 (12–25.5)	0.835
	LF (nu)	74.7 (58.3–85.1)	82.67 (66.0–87.4)	0.311
	HF (nu)	24.75 (14.9–41.4)	17.31 (12.5–33.9)	0.364
	LF/HF ratio	3.02 (1.4–5.8)	4.78 (1.9–7.0)	0.364

Abbreviations: AD, Alzheimer's disease; CG, control group; HF, high frequency; LF, low frequency; nu, normalized units; VLF, very low frequency.

Notes: *Mann-Whitney test: data expressed as median and first and third quartile values; values in bold indicate statistical significance.

**Table 3:** Heart rate variability components for the supine and orthostatic positions

	CG (n = 11)			AD group (n = 15)		
	Supine position	Orthostatic position	* *p*	Supine position	Orthostatic position	* *p*
RRi (n)	781 (775–830)	704 (689–737)	**0.003**	949 (821–1013.5)	804 (707–923)	**0.0007**
SDNN (ms)	8.5 (7.6–8.7)	8.7 (6.8–13.2)	0.656	13.5 (9.95–17.45)	11.9 (7.65–15.8)	0.125
RMSSD (ms)	7.9 (6.5–14.1)	7.5 (4.5–12.1)	0.061	12.5 (8.55–19.95)	8.2 (6.5–9.7)	**0.0008**
PNN50 (%)	0 (0–0.18)	0.00 (0.0–0.0)	0.715	0 (0–1.71)	0.00 (0.0–0.0)	**0.018**
VLF (ms ^2^ )	11 (9–19)	11 (5–21.5)	0.350	24 (12.5–54.5)	28 (11.5–50.5)	0.244
LF (ms ^2^ )	30 (22.5–42.5)	37 (21.5–82)	0.247	69 (46.5–120.5)	88 (31.5–157.0)	0.649
HF (ms ^2^ )	18 (10.5–54.5)	17 (5.0–32.5)	0.068	48 (23–114.5)	15 (12–25.5)	**0.006**
LF (nu)	52.38 (35.4–71.9)	74.7 (58.3–85.1)	**0.016**	60.67 (42.1–75.4)	82.67 (66.0–87.4)	**0.019**
HF (nu)	47.40 (27.8–63.8)	24.75 (14.9–41.4)	**0.016**	39.15 (24.5–57.7)	17.31 (12.5–33.9)	**0.019**
LF/HF ratio	1.11 (0.58–2.59)	3.02 (1.4–5.8)	**0.026**	1.55 (0.7–3.1)	4.78 (1.9–7.0)	**0.040**

Abbreviations: AD, Alzheimer's disease; CG, control group; HF, high frequency; RRi, R-R interval; LF, low frequency; nu, normalized units; PNN50, percentage of adjacent RRis with a duration difference > 50 ms; RMSSD, root mean square of the successive differences in RRis; SDNN, standard deviation of all normal-to-normal (NN) RRis; VLF, very low frequency.

Notes: *Wilcoxon test. Data expressed as median and first and third quartile values; values in bold indicate statistical significance.

The number of individuals who experienced falls in the past 3 years was also collected: falls were reported by 3 (27%) women in the CG and by 10 out of 14 (71%) AD patients. The results of the analysis of interaction involving HRV parameters and fall events are presented in Tables 4 and 5 for the supine and orthostatic positions respectively.

**Table 4:** Interaction between Heart Rate Variability components and presence of falls for supine position in time and frequency domains

	Falls (n)	CG	AD group	*p*
** Mean RRi (n) ^a^**	**0**	795.63* ± 46.58	**945.5 ± 88.13**	**0.7885**
	1	798.00 ± 86.43	925.7* ± 115.27	
Mean SDNN (ms) ^b^	0	8.85* ± 2.73	22.65* ^#^ ± 14.25	**0.0221**
	1	13.57 ± 5.85	**13.57 ± 6.13**	
Mean RMSSD (ms) ^a^	0	9.14 ± 4.26	14.18 ± 6.61	0.3103
	1	15.77 ± 9.51	14.88 ± 6.70	
Mean PNN50 (%) ^b^	0	0.05 ± 0.13	0.92 ± 1.19	0.1445
	1	2.06 ± 3.26	1.15 ± 1.70	
Mean VLF (ms ^2^ ) ^b^	0	**13.63 ± 7.09**	137.2 ± 154.6*#	0.2993
	1	16.33 ± 12.66	**29.5 ± 24.93**	
Mean LF (ms ^2^ ) ^a^	0	29.25 ± 15.84	432.5 ± 509.36	0.0588
	1	47.33 ± 30.66	85.2 ± 98.24	
Mean HF (ms ^2^ ) ^a^	0	25.25 ± 21.51	66.75 ± 59.95	0.1592
	1	115.67 ± 99.08	80.2 ± 65.47	
Mean LF (nu) ^a^	0	54.98 ± 18.93	80.43 ± 10.73	0.3259
	1	39.91 ± 33.58	47.39 ± 18.79	
Mean HF (nu) ^a^	0	44.69 ± 18.8	19.51 ± 10.7	0.3316
	1	59.94 ± 33.5	52.45 ± 18.79	
Mean LF/HF ratio ^b^	0	**1.58 ± 1.02**	6.56 ± 6.37*#	0.2165
	1	1.36 ± 1.88	**1.17 ± 0.91**	

Abbreviations: AD, Alzheimer's disease; CG, control group; HF, high frequency; RRi, R-R interval; LF, low frequency; nu, normalized units; PNN50, percentage of adjacent RRis with a duration difference > 50 ms; RMSSD, root mean square of the successive differences in RRis; SDNN, standard deviation of all normal-to-normal (NN) RRis; VLF, very low frequency.

Notes: ^a^ Analysis of variance (ANOVA) and Tukey's test. ^b^ Gamma distribution and Wald's test. *Interaction with difference between the CG and the AD group. ^#^ Interaction with difference between fall (1) and no fall (0) within group. Values in bold indicate statistical significance.

**Table 5:** Interaction between heart rate variability components and the presence of falls in the orthostatic position in the time and frequency domains

	Falls	CG	AD group	*p* *
Mean RRi (n) ^a^	0	710.5 ± 29.86	840.75 ± 156.87	0.9980
	1	699.33 ± 76.29	829.8 ± 103.94	
Mean SDNN (ms) ^b^	0	10.24 ± 4.91	16.83 ± 3.38	0.4079
	1	10.07 ± 4.08	11.12 ± 6.55	
Mean RMSSD (ms) ^a^	0	8.01 ± 4.44	10.43 ± 4.03	0.5269
	1	10.47 ± 6.57	9.75 ± 6.16	
Mean PNN50 (%) ^b^	0	0.24 ± 0.55	0.1 ± 0.2	**0.0159**
	1	**0.37 ± 0.37**	0.41 ± 0.88*	
Mean VLF (ms ^2^ ) ^b^	0	69.63 ± 60.06	188.5 ± 188.5	0.4724
	1	31.67 ± 14.36	83.4 ± 101.5	
Mean LF (ms ^2^ ) ^a^	0	27.5 ± 40.2	33 ± 21.83	0.8287
	1	47.67 ± 52.56	42.8 ± 66.6	
Mean HF (ms ^2^ ) ^a^	0	74.07 ± 15.63	85.92 ± 2.26	0.8006
	1	54.23 ± 32.41	69.88 ± 14.63	
Mean LF (nu) ^a^	0	25.67 ± 15.39	14.06 ± 2.27	0.7901
	1	45.57 ± 32.27	29.97 ± 14.55	
Mean HF (nu) ^a^	0	4.79 ± 4.25	6.24 ± 1.05	0.9272
	1	2.28 ± 2.54	3.47 ± 2.83	
Mean LF/HF ratio ^b^	0	**14.88 ± 10.34**	56 ± 8.04*#	0.7546
	1	8.67 ± 4.93	**20 ± 17.71**	

Abbreviations: AD, Alzheimer's disease; CG, control group; HF, high frequency; RRi, R-R interval; LF, low frequency; nu, normalized units; PNN50, percentage of adjacent RRis with a duration difference > 50 ms; RMSSD, root mean square of the successive differences in RRis; SDNN, standard deviation of all normal-to-normal (NN) RRis; VLF, very low frequency.

Notes: ^a^ Analysis of variance (ANOVA) and Tukey's test. ^b^ Gamma distribution and Wald's test. *Interaction with difference between the CG and the AD group. ^#^ Interaction with difference between fall (1) and no fall (0) within group. Values in bold indicate statistical significance.

The test of correlation between HRV variables and the occurrence of falls in the CG for the supine position showed a moderate correlation only with the HF (ms ^2^ ; r = 0.66195; *p* = 0.0265). In the AD group, for the supine position, the results showed a moderate correlation regarding falls and the LF/HF ratio (r = −0.625; *p* = 0.017), very LF (VLF; ms ^2^ ; r = −0.548; *p* = 0.042), LF (ms ^2^ ; r = −0.534; *p* = 0.049), LF (nu; r = −0.685; *p* = 0.0068) and HF (nu; r = 0.684; *p* = 0.0069). For the orthostatic position, no correlations were found in the CG, whereas a strong negative correlation was observed in the AD group for the LF/HF ratio (r = −0.74237; *p* = 0.0024), and moderate correlations for HF (ms ^2^ ; r = −0.524; *p* = 0.054) and LF (nu; r = 0.523; *p* = 0.055).

## DISCUSSION

Autonomic dysfunction (measured by HRV) is evident in AD patients and is a known predictor of falls in the elderly. However, the direct association between specific HRV indices and the occurrence of falls in individuals with AD-related dementia remains poorly investigated. To our knowledge, the present study is the first to assess HRV and its relationship with the occurrence of falls in AD subjects. Furthermore, the study successfully used a heart rate monitor for this assessment, demonstrating that this method is feasible and promising for the AD patient population.

In the current study, the results revealed that both groups were similar in terms of demographic, clinical, and anthropometric characteristics, except for lower cognitive performance and greater sedentary lifestyle in the AD group.

Regarding the HRV analysis, the results for the time domain in the supine position indicated lower overall HRV in the CG, likely due to aging. Although the initial frequency analysis in absolute values suggested higher sympathetic activity (LF and VLF) in AD participants, the analysis using normalized units did not sustain this LF difference as clinically relevant, suggesting that resting autonomic balance was maintained in both groups. In the orthostatic position, this distinction was not repeated, and the LF parameters (ms ^2^ or un) showed no difference between groups. Although the AD participants exhibited greater predominance of the very HF (VHF) component, this finding is generally considered to hold little physiological significance. 15

The intragroup analysis showed that the overall HRV (RRi) was significantly lower in the orthostatic position in both groups, suggesting impaired cardiovascular control due to aging. Specifically for the AD participants, the time domain data indicated a more marked impairment in parasympathetic activity, evidenced by a significant reduction in the following components: the root mean square of successive differences in RRis (RMSSD) and the percentage of adjacent RRis with a duration difference > 50 ms (pNN50). 15 In the frequency domain, both groups exhibited significant reduction in HF (nu) and increases in the LF (nu) and LF/HF ratio in the orthostatic position. These expected abnormalities were more pronounced in the AD group. Significantly, these findings appeared only in normalized units, confirming that this scale is essential to accurately capture changes in autonomic control when participants are standing, as absolute values (ms ^2^ ) failed to detect them. 22

The present investigation is one of only a few studies 12 23 24 on AD that has reported frequency domain analyses using normalized units, a key methodological step that improves the interpretability of autonomic changes.

The HRV abnormalities observed in the current study corroborate previous findings in AD regarding overall impairment. However, evidence of parasympathetic inhibition in the time domain in the orthostatic position has been seldom reported in the literature, with studies showing either no significant alteration 12 19 23 or a significant change. 25

This result supports the interpretation of autonomic dysfunction in AD, as it aligns with the robust changes observed in the frequency domain, and it helps contextualize the limitations of previous work that relied exclusively on spectral analyses of HRV, expressed solely in absolute values (ms ^2^ ).

A diagnosis of dysautonomia can serve as an early marker of dementia, helping to prevent complications that can increase the risk of hospital admissions and morbimortality. However, there is scant research 5 10 addressing the assessment of the autonomic system, particularly with respect to the risk of falls and postural hypotension in AD patients.

Studies 18 consistently show that dementia significantly increases the risk of falls in older adults, largely promoted by factors such as psychotropic drug use, hypotension, and drowsiness. In line with this, in the present study, the AD participants presented a substantially larger number of falls compared with the CG (34 versus 4 events respectively), resulting in fractures and lesions in 7 out of the 10 individuals affected.

Furthermore, a significant association was observed between the HRV and history of falls in the AD group. In the supine position, falls were associated with global HRV reductions (RRis, the standard deviation of all normal-to-normal RRis [SDNN]), as well as specific components (VLF) and reduced sympathovagal balance (LF/HF ratio). Conversely, in the orthostatic position, individuals reporting falls displayed higher parasympathetic activity (pNN50) and reduced sympathetic modulation (LF/HF ratio). The correlations observed between falls and HRV were moderate, but they critically suggest that reduced sympathetic activity and higher parasympathetic activity increase the risk of falls. By contrast, no significant correlations between HRV and falls were observed in the CG.

Dysautonomia in AD is expected, due to the influence of structures of the central nervous system on autonomic regulation, in which central cholinergic deficit has been implicated as a determining factor. 8 In addition, neuronal degeneration and tangles in AD affect the regions of the brain responsible for adrenergic neuron synthesis, impairing vasomotor function in the presence of orthostatic stress. 24 In this context, it is pertinent to address the potential interference of medications on the autonomic system.

While drugs like Beta-blockers and anticholinesterases are known to modulate cardiac autonomic control, 19 we deliberately did not exclude participants based on their use, reflecting the reality of their clinical status. 10 In the current study, the frequencies of these drugs were similar across both groups, mitigating their potential confounding effect on the group comparison. In addition, our approach is supported by studies that have shown HRV abnormalities in AD even when anticholinesterases were excluded, 25 or when there was no influence of medication on HRV results. 9

While anticholinesterase drugs can modulate (increased) parasympathetic function, the present study failed to identify this effect. We consistently observed sympathetic overactivity, which has been associated with poor cardiovascular adaptation in AD 8 and strongly suggests the presence of dysautonomia. 12

It is important to mention that dysautonomia has been strongly associated with cognitive decline, regardless of cholinergic medication, and hypertension or diabetes, 9 recognized conditions known to cause dysautonomia. A previous study 25 verified the presence of dysautonomia in dementia even after excluding diabetic patients, attributing the condition to the process of dementia progression itself. Moreover, the prevalence of these comorbidities was similar between our groups.

Concerning cognitive decline, evidence 9 24 25 26 27 suggests it can, in itself, actively influence the course of dementia. Therefore, assessing cognitive performance and autonomic function is essential to understand disease progression. In contrast, no correlation was observed between the MMSE scores and HRV variables in the current investigation.

The HRV is a non-invasive measure with potential to predict cognitive decline and the risk of death in patients with dementia, 25 including the differentiation of autonomic impairment across dementia types. 23 Incorporating the HRV into the clinical assessment of AD can effectively help identify dysautonomia and orthostatic hypotension using sympathovagal balance data. This insight enables the planning of assertive interventions to mitigate the risk of falls and other associated complications, 28 29 and it contributes to the early detection and monitoring of dementia progression. 30 Moreover, to overcome the current lack of standardization which limits HRV's clinical use, 30 heart rate monitors emerge as a viable alternative to electrocardiograms, eliminating the need for specialized clinical facilities, thus enhancing feasibility. 18 19 31 32

The current study has certain limitations that warrant discussion. The small sample size precluded an in-depth multivariate analysis on falls, the HRV, medication use, and comorbidities. However, medications for cognitive symptoms, particularly cholinergic drugs, increase the risk of syncope and falls, which is exacerbated by polypharmacy among older adults with AD. 33 Our findings suggest that autonomic dysfunction may contribute to the risk of falls in AD participants. However, given the limited sample size, these findings must be interpreted with caution. Future HRV research should incorporate data on falls, using larger cohorts, to further strengthen these results and improve risk stratification.

In conclusion, the current study showed a marked change in autonomic modulation among the patients, more pronounced for the AD participants, highlighting impaired HRV and greater cardiovascular risk. The AD group presented more fall events that interacted with HRV components, which were observed through the reduction in sympathetic and parasympathetic activities in the supine position. However, in orthostatism, the fall events were associated with higher parasympathetic activity and reduced sympathetic activity.
